# Cullin-3 proteins be a novel biomarkers and therapeutic targets for hyperchloremia induced by oral poisoning

**DOI:** 10.1038/s41598-024-59264-4

**Published:** 2024-04-13

**Authors:** Hui Duan, Na Li, Jia Qi, Xi Li, Kun Zhou

**Affiliations:** 1 Department of Emergency Medicine, Taihe Hospital, Hubei University of Medicine, Shiyan, China; 2 Department of Vascular Surgery, Taihe Hospital, Hubei University of Medicine, Shiyan, China; 3 Department of Hematology, Taihe Hospital, Hubei University of Medicine, Shiyan, China; 4 Department of Ophthalmology, Taihe Hospital, Hubei University of Medicine, Shiyan, China; 5 Department of Physical Examination Center, Taihe Hospital, Hubei University of Medicine, Shiyan, China

**Keywords:** Oral poising, Hyperchloremia, Structure prediction, Drug targets, Biomarker, Biomarkers, Diseases

## Abstract

Oral poisoning can trigger diverse physiological reactions, determined by the toxic substance involved. One such consequence is hyperchloremia, characterized by an elevated level of chloride in the blood and leads to kidney damage and impairing chloride ion regulation. Here, we conducted a comprehensive genome-wide analysis to investigate genes or proteins linked to hyperchloremia. Our analysis included functional enrichment, protein–protein interactions, gene expression, exploration of molecular pathways, and the identification of potential shared genetic factors contributing to the development of hyperchloremia. Functional enrichment analysis revealed that oral poisoning owing hyperchloremia is associated with 4 proteins e.g. Kelch-like protein 3, Serine/threonine-protein kinase WNK4, Serine/threonine-protein kinase WNK1 and Cullin-3. The protein–protein interaction network revealed Cullin-3 as an exceptional protein, displaying a maximum connection of 18 nodes. Insufficient data from transcriptomic analysis indicates that there are lack of information having direct associations between these proteins and human-related functions to oral poisoning, hyperchloremia, or metabolic acidosis. The metabolic pathway of Cullin-3 protein revealed that the derivative is Sulfonamide which play role in, increasing urine output, and metabolic acidosis resulted in hypertension. Based on molecular docking results analysis it found that Cullin-3 proteins has the lowest binding energies score and being suitable proteins. Moreover, no major variations were observed in unbound Cullin-3 and all three peptide bound complexes shows that all systems remain compact during 50 ns simulations. The results of our study revealed Cullin-3 proteins be a strong foundation for the development of potential drug targets or biomarker for future studies.

## Introduction

Hyperchloremia is an electrolyte imbalance characterized by an elevated concentration of chloride in the bloodstream. Chloride is a vital electrolyte present primarily in the extracellular fluid and closely linked to sodium^1^. Together, these ions play important roles in sustaining appropriate fluid stability, regulating blood pressure, and facilitating muscle and nerve function. Normal blood chloride levels generally fall within the range of 96 to 106 milliequivalents per liter (mEq/L). Chloride's implication as an electrolyte is underlined by its function in confirming the appropriate functioning of the body's metabolism^2^. Chloride also plays a conclusive role in enduring the body's acid–base balance. In cases of bacterial infections, there can be indirect role to electrolyte disproportions, and in severe cases, kidney function may be impacted. Hyperchloremia often arises due to underlying health issues, such as dehydration, kidney dysfunction, respiratory alkalosis, or metabolic acidosis^3,4^. Chloride supports to keep the acid and base stability in the body^4^.

Oral poisoning, specifically through the ingestion of substances that are toxic to the body, can potentially lead to hyperchloremia as part of the overall physiological response to poisoning. The development of hyperchloremia in cases of oral poisoning is dependent on several factors, including the type of toxin ingested and the extent of the poisoning. Such as some toxic substances, such as arsenic, heavy-metal, organophosphates, cholinergic toxicity, parathion, gastroenteritis etc. can cause vomiting, diarrhea, or excessive urination. These symptoms can lead to significant fluid loss from the body, resulting in dehydration and the concentration of chloride ions in the blood can increase, leading to hyperchloremia^5–7^. Certain toxins can disrupt the body's acid–base balance, which can result in metabolic acidosis leading to a surplus of acid in the body or a reduction in bicarbonate levels. To uphold electrical neutrality, chloride ions may migrate into the bloodstream as a replacement for the diminished bicarbonate ions, thus playing a role in the development of hyperchloremia in such cases^2^. Similarly, drugs or toxins can damage the kidney and dysfunction it and it may not be able to properly control chloride reabsorption, potentially leading to hyperchloremia^8,9^.

Drug-induced kidney failure accounts for a significant portion, approximately 20%, of cases of acute kidney injury, both in community and hospital settings. The prevalence is notably higher in grownup individuals underlying health conditions and are exposed to more diagnostic procedures and treatments. While clinical and demographic factors have been identified as risk factors, understanding the mechanisms of cells underlying drug-induced kidney injury caused by various toxins is complex and multifaceted. Conventional biomarkers like serum creatinine and blood urea nitrogen, which have been traditionally used for identifying drug-induced kidney injury, have limitations in terms of providing high sensitivity, specificity, and prompt detection^10^. Therefore, there is ongoing research aimed at exploring, identifying, and developing novel biomarkers for drug-induced kidney injury, primarily through the analysis of genomics data. In the current study we aimed to carry out extensive in silico analysis for prediction of potential therapeutic targets for hyperchloremia induced by oral poisoning.

## Materials and methods

**Figure 1:**
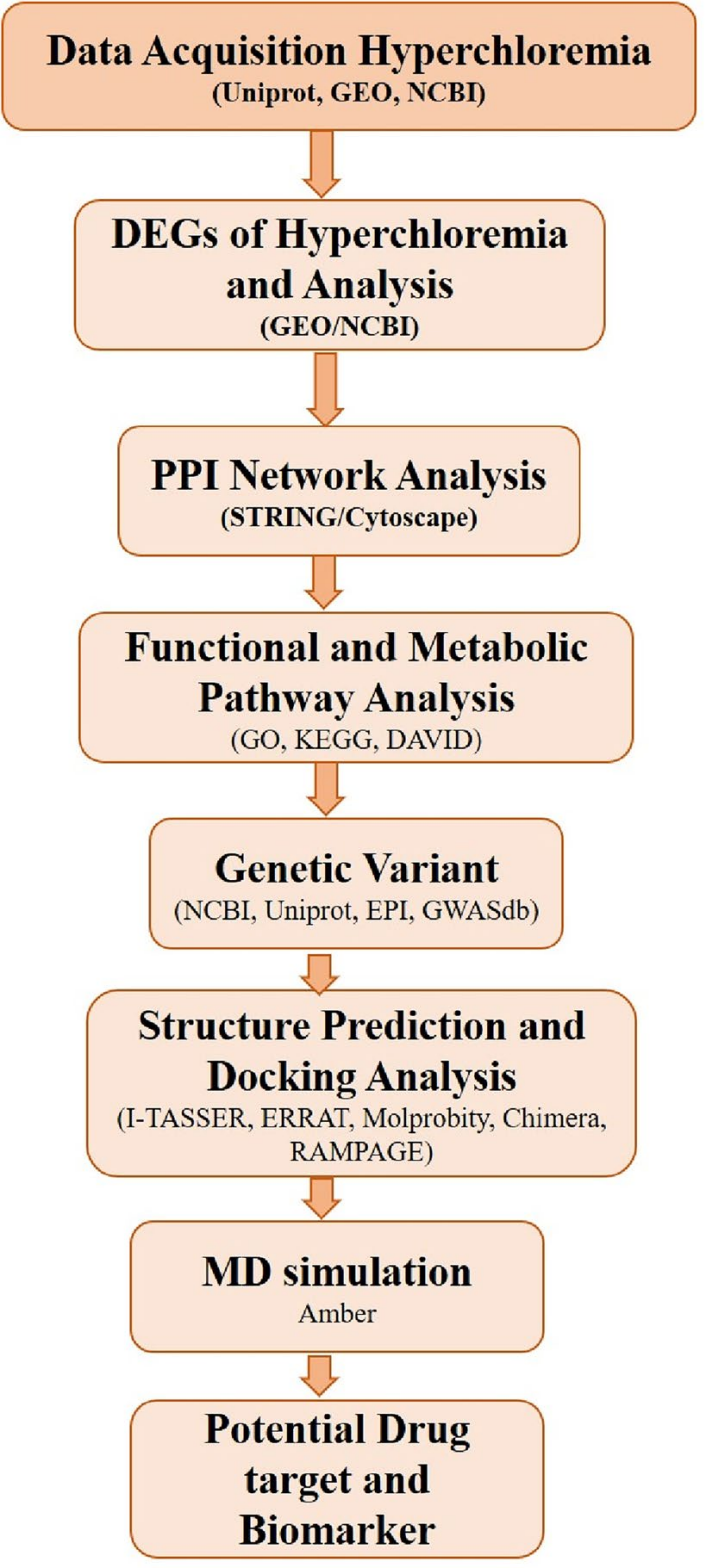
Schematic representation of the methodology carried out.

The comprehensive methodology or database utilization involved in these extensive in silico studies is presented in the form of a flowchart (Fig. 1).

### Acquisition of genomics and proteomics data associated with hyperchloremia

We conducted genome wide survey-based analysis of Hyperchloremia, oral poisoning, and associated genes. Uniport and Gene Expression Omnibus (GEO) a database of National Center for Biotechnology Information (NCBI), and Inter-Pro dataset was utilized^11,12^. Oral poisoning, hypertension, hyperkalemia, hyperchloremia, hyperchloremic metabolic acidosis, physiologic abnormalities and Human like key words were utilized. The datasets correspond to Hyperchloremia associated genes such as Kelch-like protein 3, Serine/threonine-protein kinase WNK4, Serine/threonine-protein kinase WNK1 and Cullin-3. The protein sequences, function and gene ontology of each protein were retrieved from uniport as well as EBI and Gene Ontology (GO) databases^13^.

### DEGs of hyperchloremia and analysis

Analyzing differential gene expression through data obtained by RNA sequencing (RNA-seq) has become a standard method for uncovering biological insights. Current researches aimed at processing and standardizing publicly accessible gene expression data facilitate swift and methodical re-examination. While several robust tools are available for systematically handling. In our study, we conducted an analysis of genes associated with Hyperchloremia, specifically focusing on the upregulation or downregulation of Kelch-like protein 3, Serine/threonine-protein kinase WNK4, Serine/threonine-protein kinase WNK1, and Cullin-3, using differential expression data from Gene Expression Ominibus (GEO) and studies archived within the Sequence Read Archive. *logFC* > 1 and p-value < 0.05 were regarded as the cutoff value for DEGs and GEO2R was employed to examine the data from the RNA-seq profiles. The dataset used in this study are GSE106548, GSE230608 and GSE153986.

### Recognition of protein–protein interaction network analysis

To find the interaction of hyperchloremia and its associated proteins, which are associated with conditions such as dehydration, kidney dysfunction, respiratory alkalosis, and metabolic acidosis, biological studies, pathways, and network clustering behavior were chosen. To construct a Protein–Protein Interaction (PPI) network, we used the Search Tool for the Retrieval of Interacting Genes/Proteins (STRING) database. We applied a combined score threshold of greater than 0.7 for this analysis^13^. The STRING database served as the basis for our PPI examination^14,15^. For interaction network visualization and biological pathways, we utilized Cytoscape . To identify clusters within the PPI networks, the MCODE plugin was used. We set the max depth to 2, 0.2, 0.2, and 100, degree cutoff, node score cutoff, k-core, respectively, as our threshold parameters. Furthermore, we employed the cytoHub plugin to find the hub proteins within the network by computing node scores^16^. To ensure the consistency of our findings, we studied the top 10 nodes with the highest degree using 12 different criteria such as Degree Centrality, Betweenness Centrality, Closeness Centrality, Eigenvector Centrality, PageRank, Clustering Coefficient, Node Similarity, Hub Score^17^.

### GO and KEGG pathway analysis

The Gene Ontology (GO) a treasured information source regarding gene functions^18^, and The Kyoto Encyclopedia of Genes and Genomes (KEGG) that delivers insights into the high-level functions and utilities of biological systems^19^ were used to analyze the potential functions of the DEGs proteins Kelch-like protein 3, Serine/threonine-protein kinase WNK1, Serine/threonine-protein kinase WNK4, and Cullin-3. Moreover, in order to look insights the biological functions Database for Annotation, Visualization, and Integrated Discovery (DAVID) was used which is a bioinformatic databases specifically designed for investigating the biological functions of multiple genes^20^.

### The acquisition and analysis of genetic variants in hyperchloremia-related genes

To identify the variant in Hyperchloremia-related genes such as Kelch-like protein 3, Serine/threonine-protein kinase WNK4, Serine/threonine-protein kinase WNK1, and Cullin-3, we employed various tools and databases such as MutationTaster2 and PANTHER^21,22^. The purpose of the identification of variants was to determine their potential functional significance and association with Hyperchloremia associated disease conditions and pathways such as hyperkalemia, hyperchloremia, hyperchloremic metabolic acidosis mostly caused by high inhaling of chlorine.

### Hyperchloremia associated proteins structure prediction and docking analysis

In order to look for insights into conservation and potential of small molecules for potential drug candidates, as well as to study protein–protein and protein–ligand interactions in biological systems. The structure building of the hyperchloremia associated protein was carried out. The PDB database revealed that structure of the all hyperchloremia associated proteins i.e. Kelch-like protein 3, Serine/threonine-protein kinase WNK4, Serine/threonine-protein kinase WNK1, and Cullin-3 was available with following name 4CH9, 4CHB, 4PWN and 2MYL respectively. The PDB file of these structure was submitted at ERRAT, providing the overall quality factor of respective models. Four structures were evaluated and validated using a variety of online tools, including MolProbity, for good/bad angles. According to the MolProbity results, the structures appeared reliable^23^. The physicochemical and stereochemical parameters (rotamers, outliers, stability index, and bonding patterns) and the RAMPAGE tool for poor/favored rotamers were determined to validate the predicted structures. Moreover, the structures were improved to enhance or refinement using the UCSF Chimaera 1.14.1^24^. Molecular docking was carried out using AutoDock software against the active site of the query protein with the provided ligand^25^.

### Molecular dynamic simulations

Based on the quality of the structure, protein–protein interaction analysis Cullin-3 proteins were utilized for molecular dynamic simulations. The top complexes, after protein-peptide docking, with the lowest binding energy were subjected to dynamical study using molecular dynamic simulations. Amber tools were used for this purpose to prepare files for final production run. Amber “Assisted Model Building with Energy Refinement” provides a large set of molecular mechanical force fields for bio-molecule (Proteins, Lipids, Carbohydrate) simulations which were applied through xleap program provided in Amber package^26^. First was coordinates file (.inpcrd) that contains the information about Cartesian coordinates of 3D structures of protein and peptide. Second was topology file (.prmtop) that contained information about atoms, bonds, angles and molecules present. For preparation of input files leaprc.ff14SB force field and TIP3P water model were used to prepare all systems. The next step in system preparation was loading the PDB file of complex (Protein-Peptide) then charge was checked on the system. After equilibration, the system is ready for MD simulations. The final step of simulation was to run simulations in the production phase for desired time. During the production run conformational changes that took place after every 2 fs time step were saved in the form of trajectories in DCD file, produced by NAMD. For protein and all the complexes this step consists of 25,000,000 steps (50 ns). Data produced from 50 ns MD simulation was further subjected for analysis.

## Results and discussion

### Hyperchloremia and associated gene and proteins

Predicting genes and proteins associated with hyperchloremia involves understanding the underlying molecular mechanisms and genetic factors contributing to the condition. By using literature review, GO, and Database searches, we retrieved four genes from NCBI and their four protein products from Uniport databases. Based on the literature, in silico analysis revealed that four proteins encoded the function and association with Hyperchloremia and named as Kelch-like protein 3, Serine/threonine-protein kinase WNK4, Serine/threonine-protein kinase WNK1, and Cullin-3. The name of these proteins, IDs and disease association is described in detail in Table 1.

**Table 1:** List of hyperchloremia associated proteins.

Uniprot ID	Name of protein	Disease
Q9UH77	Kelch-like protein 3	A disorder characterized by severe hypertension, hyperkalemia, hyperchloremia, hyperchloremic metabolic acidosis, and correction of physiologic abnormalities by thiazide diuretics. PHA2D inheritance is autosomal dominant or recessive
Q96J92	Serine/threonine-protein kinase WNK4	An autosomal dominant disorder characterized by hypertension, hyperkalemia, hyperchloremia, mild hyperchloremic metabolic acidosis, and correction of physiologic abnormalities by thiazide diuretics
Q96J92	Serine/threonine-protein kinase WNK1	An autosomal dominant disorder characterized by severe hypertension, hyperkalemia, hyperchloremia, mild hyperchloremic metabolic acidosis in some cases, and correction of physiologic abnormalities by thiazide diuretics
Q9H4A3	Serine/threonine-protein kinase WNK1	An autosomal dominant disorder characterized by severe hypertension, hyperkalemia, hyperchloremia, mild hyperchloremic metabolic acidosis in some cases, and correction of physiologic abnormalities by thiazide diuretics
Q13618	Cullin-3	An autosomal dominant disorder characterized by severe hypertension, hyperkalemia, hyperchloremia, hyperchloremic metabolic acidosis, and correction of physiologic abnormalities by thiazide diuretics

Presently, hyperchloremia does not manifest specific symptoms, but it can be influenced by various factors leading to the loss of fluid which is electrolyte-free, hypotonic fluid loss, or enhanced sodium chloride intake. These factors include conditions such as vomiting, diarrhea, increased sodium chloride consumption, diuretic usage, renal issues, and diabetes. It's important to differentiate hyperchloremia and its associated genes from hyperchloremic metabolic acidosis, which exhibits two main variations: a decrease in bicarbonate levels and blood pH, along with a rise in blood chloride levels. Individuals experiencing hyperchloremic metabolic acidosis are usually prone to developing hyperchloremia^27–29^.

### Differential gene expression analysis of hyperchloremia genes

Transcriptomic data obtained by various searches i.e. in silico analysis of GEO database involving Serine/threonine-protein kinase WNK1, Cullin-3, KLHL and Serine/threonine-protein kinase WNK4 protein indicates that no experimental data available exhibiting oral poisoning, hyperchloremia, or metabolic acidosis or drug inhaling. So kidney, renal familiar or metabolic acidosis dataset were searched for quantitative expression analysis of Hyperchloremia genes in response to various drugs or stress (Table 2). As mentioned in methodology, those Serine/threonine-protein kinase WNK1, Cullin-3, KLHL and Serine/threonine-protein kinase WNK4 genes were chosen whose *logFC* > 1 and p-value < 0.05 as the cutoff value for DEGs. Results revealed that hyperchloremia genes indeed showed a differential expression compared to their control.

**Table 2:** Differential gene expression analysis of hyperchloremia associated genes.

Uniprot ID	Gene Name/ID	Name of protein	Gene expression role reported by GEO
Q9UH77	KLHL3/26249	Kelch-like protein 3	Under homeostatic conditions, NRF2 is repressed by Kelch-like ECH-associated protein 1 (KEAP1)
Q96J92	WNK4/65266	Serine/threonine-protein kinase WNK4	WNK4—Akt inhibitor MK2206 effect on influenza H1N1 infection of non-small cell lung cancer line
Q9H4A3	WNK1/65125	Serine/threonine-protein kinase WNK1	WNK4 perform same function as WNK1
Q13618	CUL3/8452	Cullin-3	A group of proteins, play a significant role in determining which proteins are targeted for degradation by the E3 ligase complexes, serving as scaffold proteins

Literature analysis revealed the importance of Serine/threonine-protein kinase WNK1, Cullin-3, KLHL and Serine/threonine-protein kinase WNK4 genes. Such as in various bacterial or viral infection such as H1N1, Kelch-like ECH protein leads to hyperactivation of the antioxidant program downstream of the nuclear factor erythroid 2-like 2 (NRF2) transcription factor, and this hyperactivation is associated with a poor prognosis^30^. Moreover, recent findings indicate that the malfunctioning disposal system responsible for managing cellular proteins, known as the ubiquitin–proteasome system (UPS), perform a crucial role to the development of various abnormalities, such as cancer and neurodegenerative diseases.

There are a rising evidences that points to irregular regulation of E3 ubiquitin ligases, which are vital components of the UPS, contributing to uncontrolled cell growth, genetic instability, and the onset of cancer. This is because these ligases, along with their targets, are key players in regulating processes like cell cycle progression, gene transcription, and signal transduction. Among these components, the cullins, a group of proteins, play a significant role in determining which proteins are targeted for degradation by the E3 ligase complexes, serving as scaffold proteins^31,32^. To date, seven distinct members of the cullin family have been identified^32^. The data of these genes and proteins is described in Table 2. The KLHL gene family encodes a set of proteins typically categorized by the presence of a BACK domain, BTB/POZ domain, and five to six Kelch motifs). KLHL genes are occupied in various Mendelian diseases and have also been related to cancer^31^.

Mutations in human genes such as WNK1 (With no K [lysine] protein kinase-1) and the related protein kinase WNK4 are accountable for Gordon's hypertension syndrome^31^. Moreover, the mutations in numerous genes triggered familial hyperkalemic hypertension. The most severe form of familial hyperkalemic hypertension outcomes from mutations in CUL3, which encodes CUL3 (Cullin 3), a scaffold protein within an E3 ubiquitin ligase complex accountable for tagging substrates for proteasomal degradation^32^.

### Protein–protein interaction network analysis

**Figure 2:**
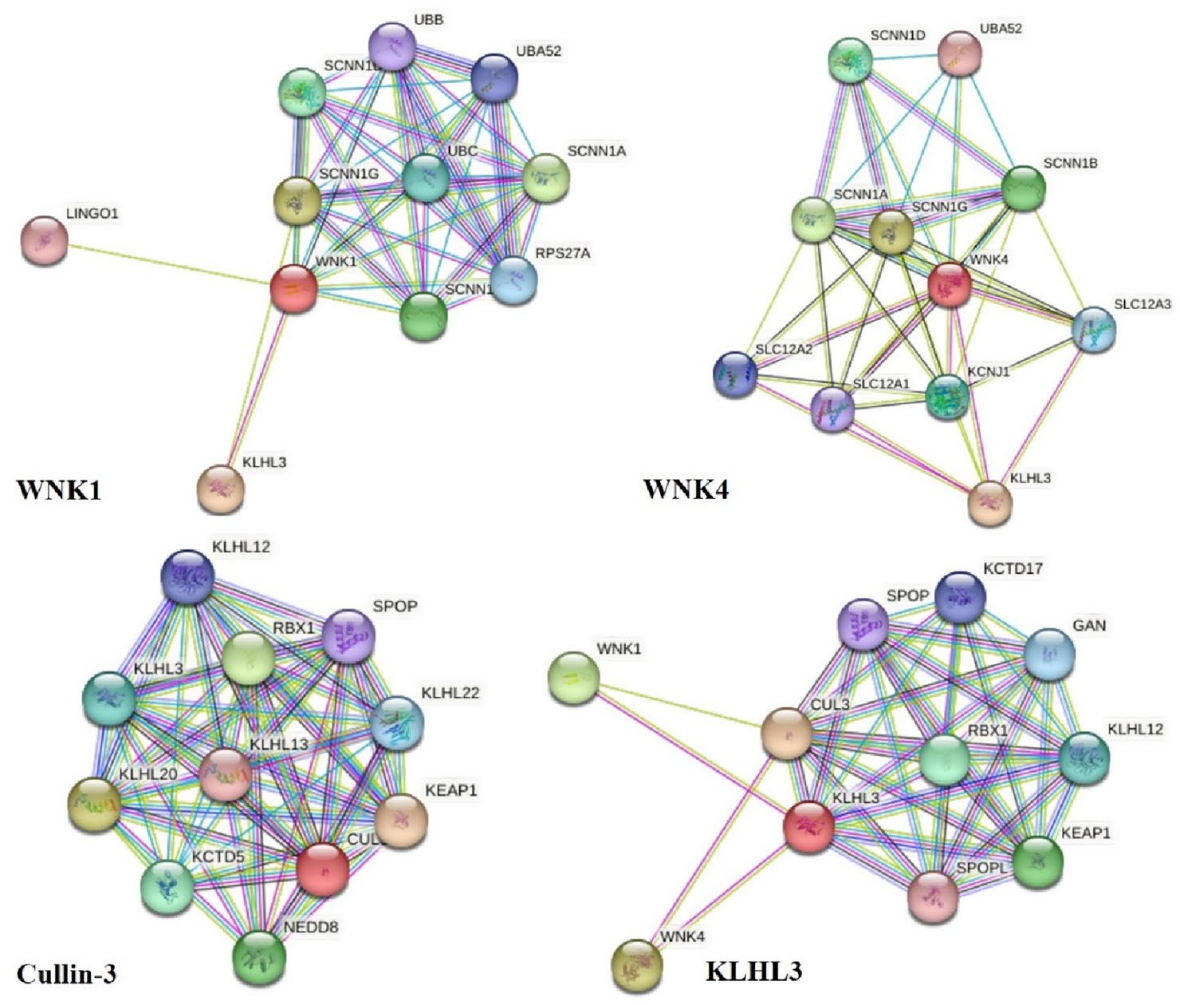
The protein–protein interaction network analysis of WNK1, WNK4, Cullin-3 and KLHL-3.

**Figure 3:**
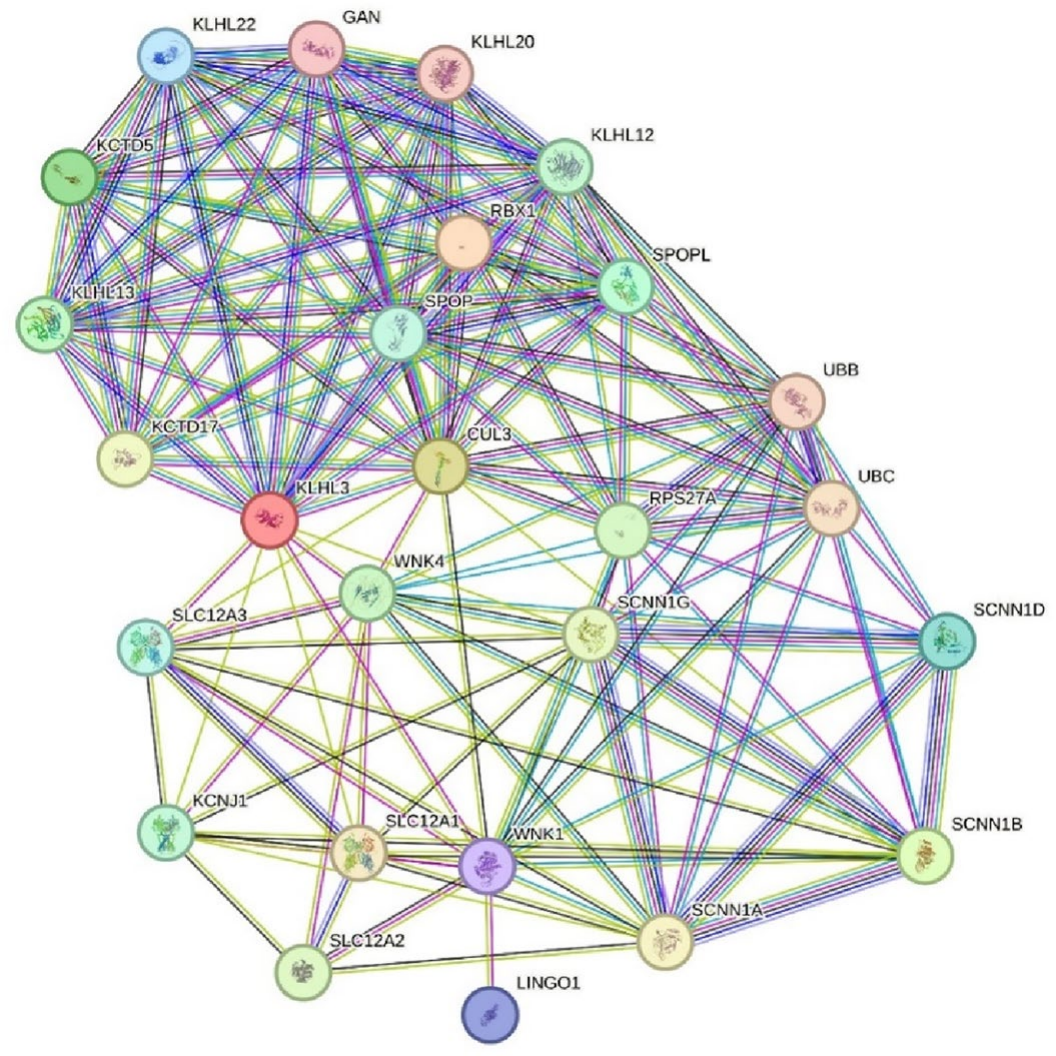
Merged protein–protein interaction network analysis of the KLHL-3, WNK4, WNK3, Cullin-3 interacted nodes.

We utilized the STRING protein databases to build a Protein–Protein Interaction (PPI) network. To evaluate the interaction consistency within the network, each edge was assigned a score as its weight. We chosed PPIs with a confidence score exceeding 0.7 to ensure the interactions' quality and to reduce the occurrence of false-positive results. The PPI data revealed that CUL3 protein encompasses 9 nodes, KLHL3 10 nodes, WNK4 encodes 9 and WNK1 encodes 8 nodes (Fig. 2). To look in depth and interaction of each protein, a new network with 35 nodes and 302 edges following merging these networks were built (Fig. 3). CUL3 was the exceptional proteins with maximum connection of 18 nodes following KLHL3 with 14 nodes indicating CUL3 seems to be potential hub proteins for the hyperchloremia associated imbalance.

The CUL3 gene provides instructions for the synthesis of a protein known as cullin-3. This particular protein is integral to the ubiquitin–proteasome system, responsible for breaking down unwanted proteins within cells. Acting as the cell's quality control mechanism, the ubiquitin–proteasome system eliminates damaged, misfolded, and surplus proteins. Cullin-3 serves as a central component of a complex called an E3 ubiquitin ligase. E3 ubiquitin ligases attach molecules called ubiquitin to proteins, marking them for degradation by specialized cell structures known as proteasomes. Moreover, the ubiquitin–proteasome system regulates numerous vital cellular processes by modulating the levels of proteins involved in them. E3 ubiquitin ligases containing cullin-3 target a diverse array of proteins that partake in various functions, including cell proliferation and division^33–36^.

Additionally, these ligases tag proteins associated with blood pressure regulation, such as WNK1 and WNK4, which stem from the WNK1 and WNK4 genes. Through the modulation of WNK1 and WNK4 levels, cullin-3 contributes to the regulation of blood pressure^37^. So, the prediction of CUL3 to be potential hub proteins corroborated with literature and seems to be potential targets as Biomarker or drug targets.

### The analysis of genetic variants in hyperchloremia-related genes

The results revealed that Kelch-like protein 3 harbors 383 various variant but among them harbors 42 pathogenic and likely pathogenic variants.

Pseudohypoaldosteronism (PHA) type disease. Serine/threonine-protein kinase WNK4 harbor 1229 reported variant and among them only 5 were reported as pathogenic variant (Table S2). These pathogenic variants also lead to PHA disease. Serine/threonine-protein kinase WNK1 harbors 1894 variant while 12 were the only variant which encodes pathogenic features such as PHA disease (Table S3). Cullin-3 harbor 391 variant and was 25 were predicted as pathogenic (Table S4). Except few such as S > * which is stop gained mutant encodes neurodevelopmental disorder with autism and majority of the variant leads to Pseudohypoaldosteronism (PHA) disease (Table 3).

**Table 3:** Variant of Hyperchloremia associated proteins and their reported pathogenic function.

Uniprot ID	Name of protein	Change	Description	AA
Q9UH77	Kelch-like protein 3	M > V	PHA2D	Aug/Gug(−), Met/Val
		A > E	PHA2D	gCg/gAg(−), Ala/Glu
		E > A	PHA2D	gAg/gCg(−), Glu/Ala
		T > A	PHA2D	Acu/Gcu(−), Thr/Ala
		Q > *	PHA2D	Cag/Uag(−), Gln/Ter
		C > F	PHA2D	uGc/uUc(−), Cys/Phe
		R > G	PHA2D	Cgu/Ggu(−), Arg/Gly
		R > *	PHA2D	Cga/Uga(−), Arg/Ter
		L > missing	PHA2D	L > missing
		Q > R	PHA2D	cAg/cGg(−), Gln/Arg
		F > C	PHA2D	uUc/uGc(−), Phe/Cys
		R > I	PHA2D	aGa/aUa(−), Arg/Ile
		A > V	PHA2D	gCa/gUa(−), Ala/Val
		R > Q	IGD	cGg/cAg(−), Arg/Gln
		V > M	PHA2D	Gug/Aug(−), Val/Met
		R > W	PHA2D	Cgg/Ugg(−), Arg/Trp
		R > Q	PHA2D	cGg/cAg(−), Arg/Gln
		R > W	PHA2D	Cgg/Ugg(−), Arg/Trp
		L > P	PHA2D	cUg/cCg(−), Leu/Pro
		A > V	PHA2D	gCa/gUa(−), Ala/Val
		S > L	PHA2D	uCg/uUg(−), Ser/Leu
		P > L	PHA2D	cCg/cUg(−),Pro/Leu
		M > T	PHA2D	aUg/aCg(−), Met/Thr
		R > Q	PHA2D	cGg/cAg(−), Arg/Gln
		R > W	PHA2D	Cgg/Ugg(−), Arg/Trp
		S > N	PHA2D	aGc/aAc(−), Ser/Asn
		S > G	PHA2D	Agu/Ggu(−), Ser/Gly
		S > N	PHA2D	aGu/aAu(−), Ser/Asn
		W > *	PHA2D	uGg/uAg(−), Trp/Ter
		W > *	PHA2D	ugG/ugA(−), Trp/Ter
		A > T	PHA2D	Gcc/Acc(−), Ala/Thr
		H > Y	PHA2D	Cau/Uau(−), His/Tyr
		G > V	PHA2D	gGg/gUg(−), Gly/Val
		P > T	PHA2D	Ccu/Acu(−), Pro/Thr
		V > I	PHA2D	Guu/Auu(−), Val/Ile
		R > C	PHA2D	Cgc/Ugc(−), Arg/Cys
		R > H	PHA2D	cGc/cAc(−), Arg/His
		N > K	PHA2D	aaC/aaA(−), Asn/Lys
		S > L	PHA2D	uCg/uUg(−), Ser/Leu
		Y > C	PHA2D	uAc/uGc(−), Tyr/Cys
		R > W	PHA2D	Cgg/Ugg(−), Arg/Trp
Q96J92	Serine/threonine-protein kinase WNK4	E > G	PHA2B	gAg/gGg(+), Glu/Gly
		P > L	PHA2B	cCa/cUa(+ , Pro/Leu
		E > K	PHA2B	Gag/Aag(+), Glu/Lys
		D > A	PHA2B	gAc/gCc(+), Asp/Ala
		D > H	PHA2B	Gac/Cac(+), Asp/His
		Q > E	PHA2B	Cag/Gag(+), Gln/Glu
		K > E	PHA2B	Aag/Gag(+), Lys/Glu
		R > C	PHA2B	Cgc/Ugc(+), Arg/Cys
Q9H4A3	Serine/threonine-protein kinase WNK1	D > *missing*	PHA2C	Arg/Ter, Stop
		Q > *missing*	PHA2C	
		M > *missing*	PHA2C	
		S > *		
		R > *		
Q13618	Cullin-3	R > *missing*		
		Y > C	PHA2D	
		L > *missing*	PHA2E	
		S > *		Ser/Ter
		S > L	PHA2E	uCa/uUa(−),
		A > G	PHA2E	
		R > *		Termination
		M > *		Meth/Ter
		I > *missing*		Framshift
		V > A		gUa/gCa(−), Val/Ala
		D > *missing*		Framshift
		D > G	PHA2E	gAu/gGu(−)
		DS > *	IGD	Stop gained
		K > R	PHA2E	aAg/aGg(−), Lys/Arg
		G > D	NK-cell	gGu/gAu(−), Gly/Asp
		S > *missing*		Frameshift
		R > *		Cga/Uga(−), Stop
		Q > *	IGD	Cag/Uag(−), stop gained

Overall it was PHA, which was the key variant predicted in all hyperchloremia associated proteins PHA is a collection of diverse disorders involving electrolyte metabolism. These conditions are marked by a seeming lack of response or resistance in the renal tubules to aldosterone's effects. PHA is evident through symptoms such as elevated potassium levels (hyperkalemia)^38^, a usual glomerular filtration rate (GFR) and metabolic acidosis. Depending on the specific condition within this syndrome, individuals may experience volume depletion or hypervolemia, renal salt excretion or retention, low blood pressure or high blood pressure, and varying levels of renin and aldosterone, which can be elevated, normal, or reduced^39,40^. These findings showed that PHA associated variants are the main being controlled by these genes and could be potential to target for biomarker and drug discovery. Similarly, the presence of Immunoglobulin D (IgD) as an antibody isotype constitutes approximately 1% of the proteins found in the plasma membranes of immature B-lymphocytes. It typically co-exists with another cell surface antibody known as IgM. Secreted IgD appears to play a role in enhancing mucosal homeostasis and immune surveillance by providing myeloid effector cells like basophils and mast cells with IgD antibodies that are reactive against mucosal antigens, including both commensal and pathogenic microbes^41,42^.

The identification of the IgD variant has illuminated the crucial role of hyperchloremia, specifically involving the Cullin-3 protein, in influencing neurological function. This association is particularly relevant in the context of an autosomal dominant disorder characterized by severe hypertension, hyperkalemia, and, in some instances, mild hyperchloremic metabolic acidosis. Physiological abnormalities associated with this disorder can often be corrected by thiazide diuretics.

### GO and KEGG pathway analysis

We conducted an integrated analysis of the KEGG pathway and GO data. The GO analysis are described in Table 4 where it is further confirmed that Kelch-like protein 3 is associated with ubiquitin-like protein transferase activity like function, Serine/threonine-protein kinase WNK4 and 1 is associated with Protein serine/threonine kinase activity and Cullin-3 protein is associated with Cullin family protein binding. Cullin-3 is a protein that plays a critical role in the ubiquitin–proteasome system leads to regulating the degradation of proteins in eukaryotic cells. In the network structure, each node represents a human protein, and the links connecting these nodes are assigned weight values that indicate the degree of biological similarity between the processes involving these four proteins.

**Table 4:** Gene ontology analysis.

Name of protein	Term	Annotation Score	Annotation term
Kelch-like protein 3	Molecular Function	1,431,208	ubiquitin-like protein transferase activity
	Cellular Components	12	Kelch-containing forming a regulatory complex
	Biological Process	2307	Toll-like receptor 3 signaling pathway
Serine/threonine-protein kinase WNK4	Molecular Function	3,234,192	Protein serine/threonine kinase activity
	Cellular Components	291, 646	Serine/threonine protein kinase complex
	Biological Process	21	Calcium ion homeostasis
Serine/threonine-protein kinase WNK1	Molecular Function	3,234,192	Protein serine/threonine kinase activity
	Cellular Components	291,646	Serine/threonine protein kinase complex
	Biological Process	21	Calcium ion homeostasis
Cullin-3	Molecular Function	46,545	Cullin family protein binding
	Cellular Components	331,186	Cullin-RING ubiquitin ligase complex
	Biological Process	30	Protein deneddylation

**Figure 4:**
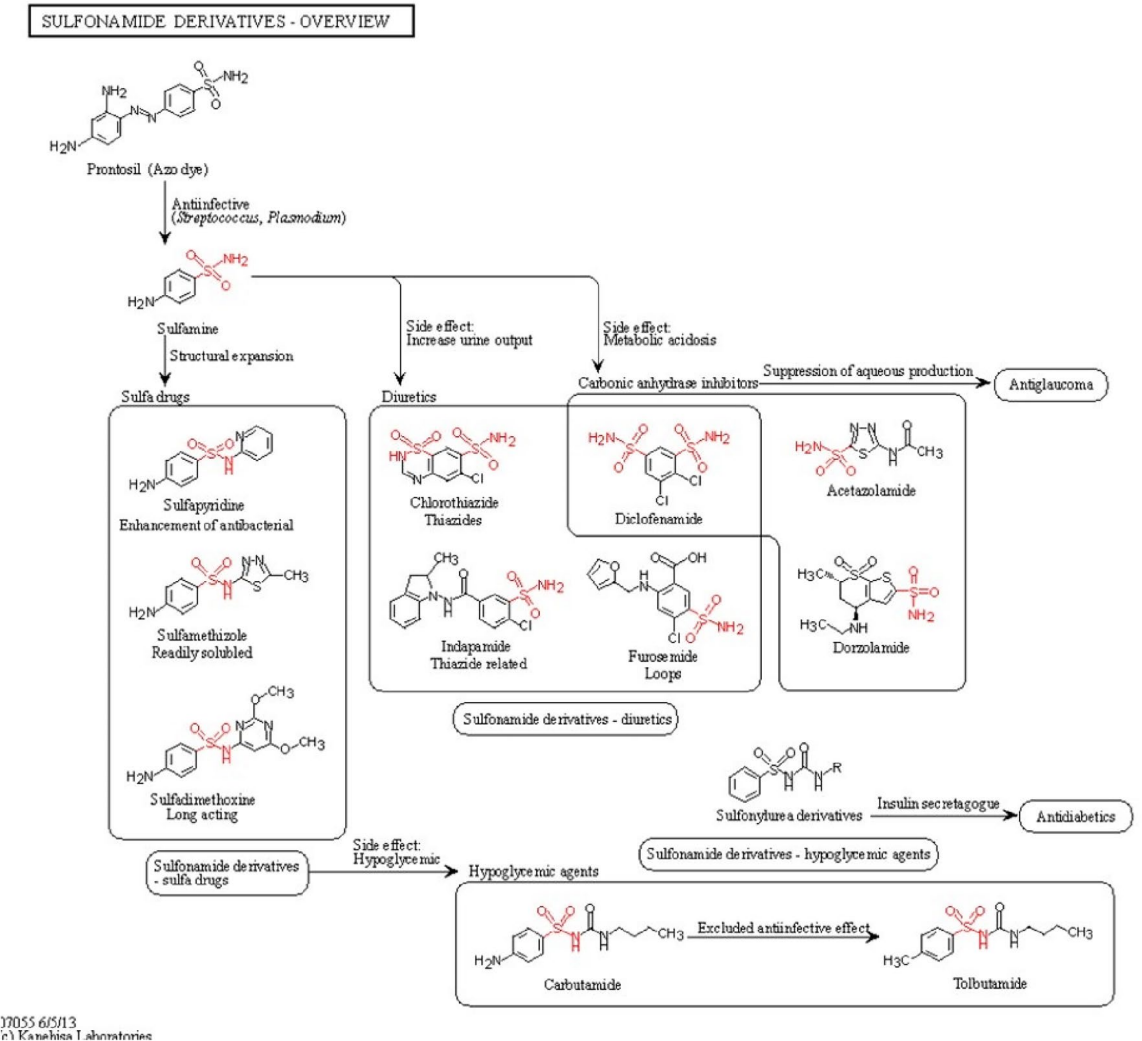
Functional enrichment analysis of the derivatives of Cullin-3 proteins.

The overview of the metabolic pathway of proteins Cullin-3 (Fig. 4) revealed that derivative is Sulfonamide which play role in increasing urine output, and metabolic acidosis resulted in the hypertension^44^. Overall pathway analysis of all the hyperchloremia associated proteins revealed the association with metabolic acidosis which is a sign of fundamental pathology. The concentration of hydrogen ions is influenced by factors such as acid production, acid ingestion, acid excretion, as well as renal and gastrointestinal bicarbonate losses.

### Hyperchloremia associated proteins structure and docking analysis

The 3D structures of four proteins were present on the Protein Data Bank (PDB) in complexes. Their 3D structures were separated from complexes and used for further study. Protein structures were assessed using the MolProbity, Rampagetool, minimized and refined via the UCSF Chimera.

The MolProbity results suggested that the structures were quite reliable. The Rampage tool showed poor/favored rotamers. Physicochemical and stereo-chemical properties (rotamers, outliers, stability index, and bonding patterns) were calculated to validate the predicted structures. The assessment results suggested that the structures of Kelch-like protein 3, Serine/threonine-protein kinase WNK4, Serine/threonine-protein kinase WNK1, and Cullin-3 were quite reliable for molecular docking to identify protein binding sites and shared features.

**Figure 5:**
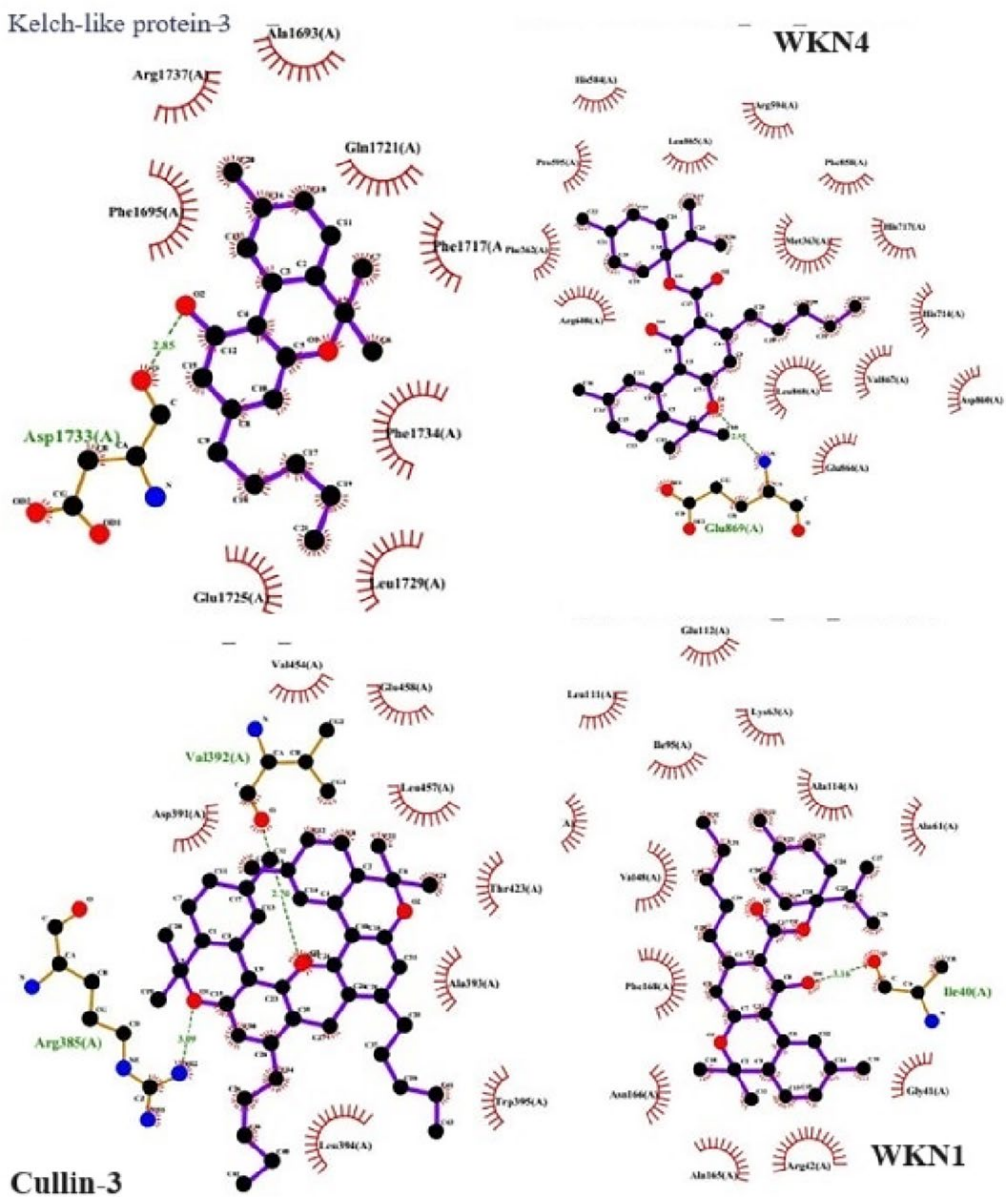
The molecular docking analysis of Cullin-3, WKN1, WKN4 and Ketch-Like proteins.

Molecular docking results analysis showed that had a binding energy of -589.61 kJ/mol for Kelch-like protein 3, WNK1/WNK4 with a binding score of -452.40 kJ/mol and Cullin-3 with a binding energy of -254.72 kJ/mol, respectively. For binding interaction analysis, different tools were used such as LigPlus and PDBSum database. The results revealed that residues with a best interaction were observed and compared with each other such as Kelch-like protein 3, Serine/threonine-protein kinase WNK4, Serine/threonine-protein kinase WNK1, and Cullin-3 M. Top 1 (Cullin-3) proteins was selected based on its highest binding energies score, and protein–protein interactions using LigPlot. These results showed their binding sites, binding distance, and hydrophobic residues (Fig. 5).

### MD simulation results and analysis

RMSD plots helps to analyze the stability of a protein or complex with respect to time and also indicates the conformational changes and displacements occur in them.

**Figure 6:**
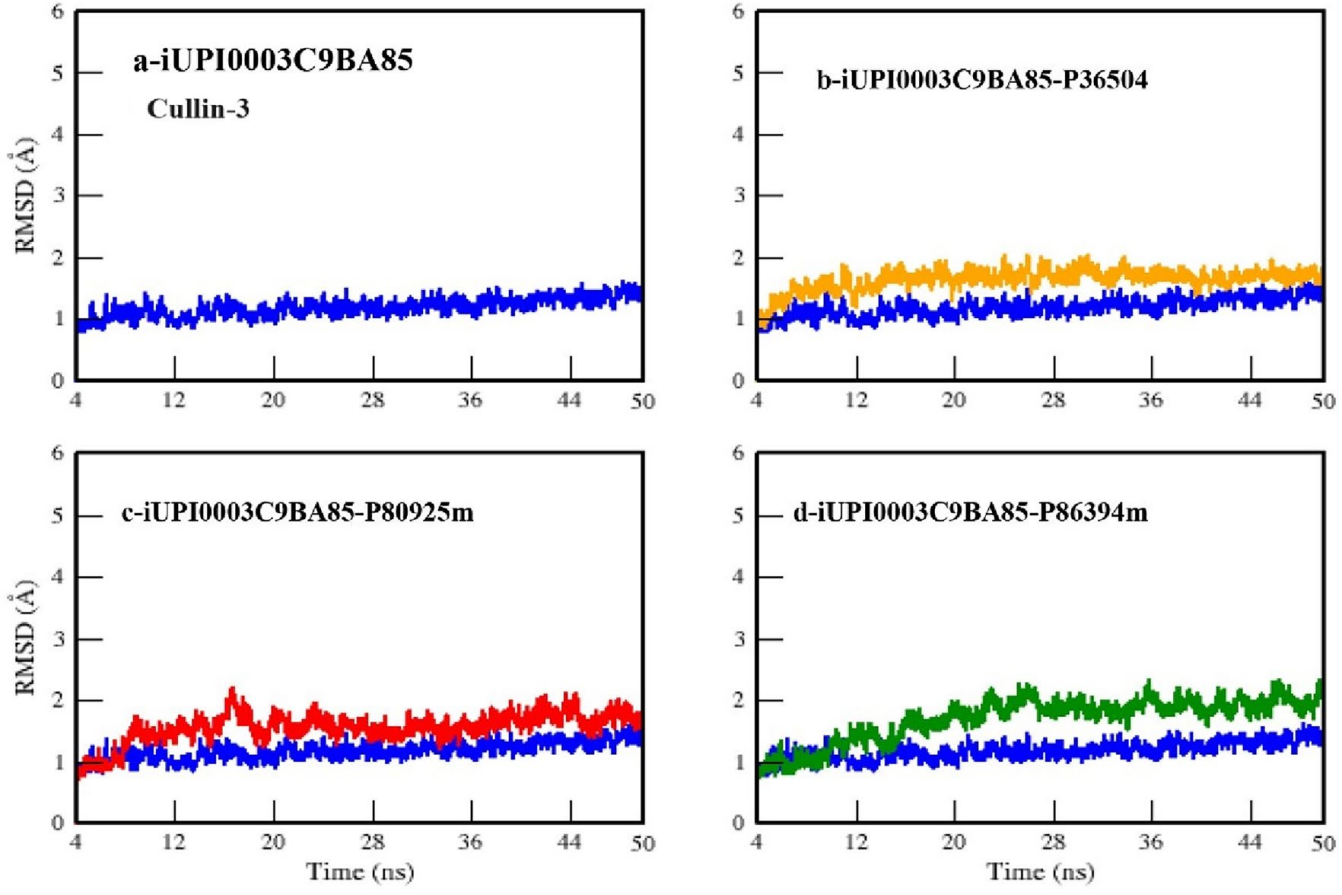
RMSD Plot of UPI0003C9BA85 (blue) (Cullin-3) with its three complexes (**a**) UPI0003C9BA85, (**b**) UPI0003C9BA85-P36504 complex, (**c**) UPI0003C9BA85-P80925m complex, (**d**) UPI0003C9BA85-P86394m.

Figure 6 shows the RMSD plots of UPI0003C9BA85 (Cullin_3) and its three complexes with starting 1000 frames (3 ns) excluded. In this plot of UPI0003C9BA85 (blue), we can notice the average RMSD value ranges between 1 and 1.5 Å with almost no deviation. This value indicates the stability of Cullin-protein throughout the simulations. The RMSD plot of UPI0003C9BA85-P36504 complex (Fig. 6b) shows average value of RMSD from 1 to 1.8 Å with almost no deviation. The RMSD plot of UPI0003C9BA85-P80925m complex (Fig. 6c) ranges from 1 to 1.5 Å and where it shows deviation from 6 to 8 ns and 16 to 18 ns, and then it becomes stable.

A little deviation is also observed at 20 and 23 ns and then was seems to be stable throughout the simulation. The RMSD plot of UPI0003C9BA85-P86394m complex (Fig. 6-d) ranges from 1 to 2 Å. It is observed that the RMSD value slightly increasing with the passage of time from 12 to 20 ns but after that it become stable up to 50 ns. Overall, we can say that that peptides like P36504 and P80925m and P86394m did not cause any large structural changes throughout simulations. All the complexes show maximum stability throughout simulations of 50 ns. RMSF was calculated to analyze the atomic fluctuations in residues and to identify the fluctuating regions in protein and complexes. Lower RMSF values indicate stable residues whereas high RMSF value shows flexibility and fluctuations of the respective residues.

**Figure 7:**
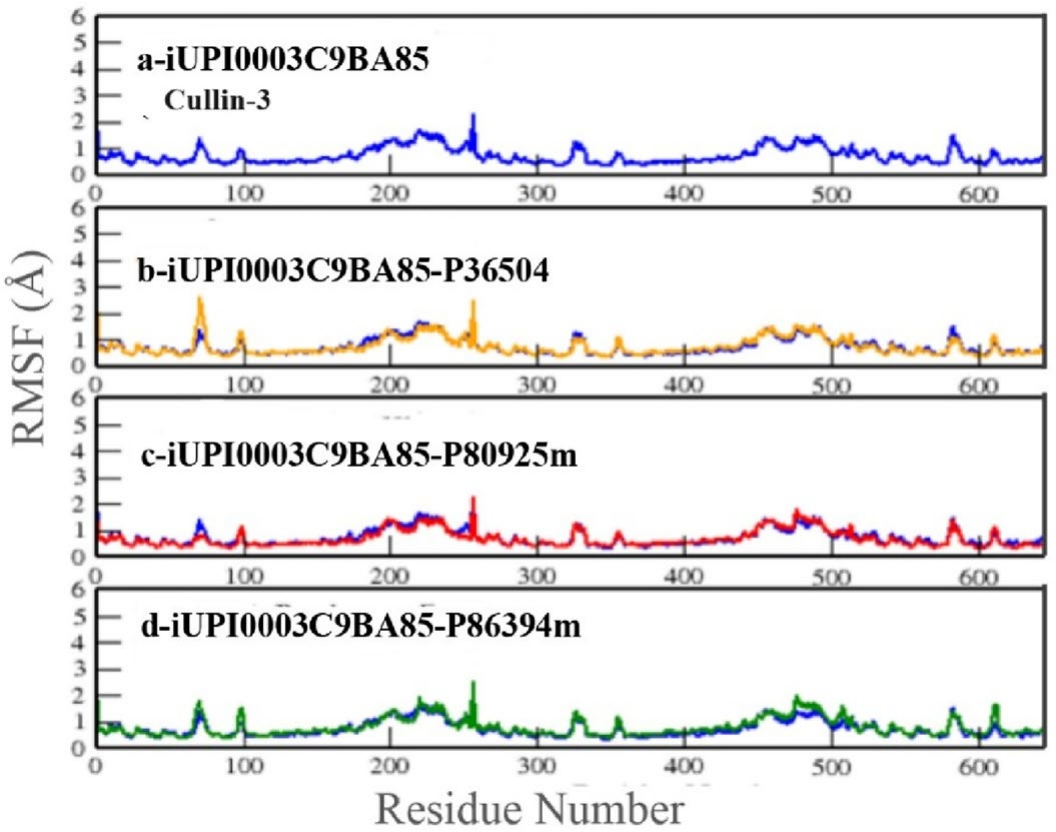
RMSF Plot of UPI0003C9BA85 (blue) (Cullin-3) with its three complexes (**a**) UPI0003C9BA85, (**b**) UPI0003C9BA85- P36504 complex. (**c**) UPI0003C9BA85-P80925m complex, (**d**) UPI0003C9BA85P86394m.

Figure 7 shows the RMSF plot of UPI0003C9BA85 and its three complexes. No major peak was observed for RMSF of UPI0003C9BA85 (Fig. 7a). RMSF graphs of UPI0003C9BA85-P36504 (Fig. 7b), UPI0003C9BA85-P80925m (Fig. 7c), and UPI0003C9BA85-P86394m (Fig. 7-d) were compared and all of them show almost same pattern where no higher peaks were noted. For all three complexes the average value of RMSF was ranges from 1 to 2 Å. present. To measure the compactness of protein and peptide structures, the Radius of Gyration (ROG) was calculated. During simulations the value of radius of gyration remains steady shows protein or complex stability however, if unfolding occurs in protein the value does not remain same or fluctuates with respect to time during simulations.

**Figure 8:**
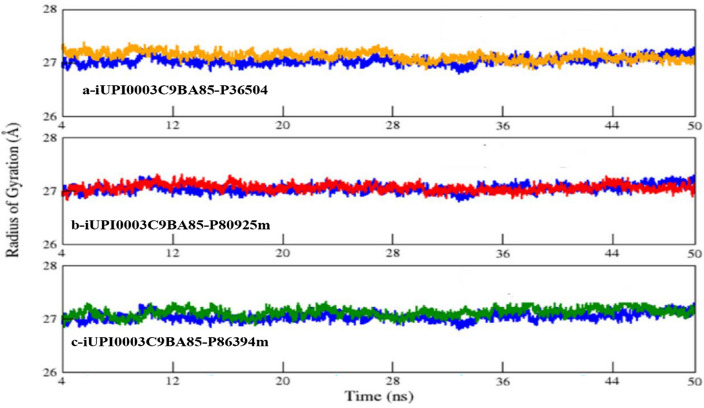
ROG Plot of UPI0003C9BA85 (blue) (*Cullin-3*) with its three complexes (**a**) UPI0003C9BA85-P36504 complex, (**b**) UPI0003C9BA85-P80925m complex, (**c**) UPI0003C9BA85-P86394m.

Figure 8 shows the ROG plot of UPI0003C9BA85 (Cullin-3) and its three complexes. It has been observed that the value of ROG for UPI0003C9BA85-P36504 (Fig. 8a), UPI0003C9BA85-P80925m (Fig. 8b), and UPI0003C9BA85-P86394m (Fig. 8c) complexes remain steady indicating that the complex remains stable throughout the 50 ns simulations and no unfolding event is observed. No major variations were observed in unbound Cullin-3 and all three peptide bound complexes shows that all systems remain compact during 50 ns simulations.

## Conclusion

Through a comprehensive genome-wide analysis concentrating on genes related to oral poisoning and hyperchloremia, we investigated the metabolic pathway involving proteins like Cullin-3. This examination unveiled that Cullin-3's derivative is Sulfonamide, which contributes to structural expansion, heightened urine output, and the development of metabolic acidosis, leading to hypertension. Furthermore, the analysis of molecular docking and simulation results demonstrated that Cullin-3 proteins exhibited the most substantial binding energy scores and stability. This finding suggests that Cullin-3 proteins hold great promise as promising candidates for the development of potential drug targets or biomarkers in future studies.

### Supplementary Information

Supplementary Table S1.

Supplementary Table S2.

Supplementary Table S3.

Supplementary Table S4.

## Data Availability

The datasets analyzed during the current study are available at Uniprot under following link (Hyperchloremia in UniProtKB search (4) | UniProt).
